# Maternal mental health in Amhara region, Ethiopia: a cross-sectional survey

**DOI:** 10.9745/GHSP-D-14-00119

**Published:** 2014-12-02

**Authors:** Joy Noel Baumgartner, Angela Parcesepe, Yared Getachew Mekuria, Dereje Birhanu Abitew, Wondimu Gebeyehu, Francis Okello, Dominick Shattuck

**Affiliations:** a FHI 360, Washington, DC, USA. Now with Duke Global Health Institute at Duke University, Durham, NC, USA; b University of North Carolina at Chapel Hill, Gillings School of Public Health, Chapel Hill, NC, USA; c FHI 360, Addis Ababa, Ethiopia; d Bahir Dar University, Bahir Dar, Ethiopia; e Amhara Regional Health Bureau, Bahir Dar, Ethiopia; f FHI 360, Addis Ababa, Ethiopia. Now with Abt Associates, SHOPS Project, Bethesda, MD; g FHI 360, Livelihoods and Food Security Technical Assistance II (LIFT II), Durham, NC, USA

## Abstract

**Background:**

Postpartum common mental disorders (CMD) such as depression and anxiety are increasingly recognized for their burden in low-resource countries such as Ethiopia. However, the magnitude of postpartum CMD in Ethiopia is not well-established. This short report describes the mental health status of women who had given birth in the last 24 months in the Amhara region of Ethiopia.

**Methods:**

A cross-sectional survey was conducted among 1,319 women aged 15–49 years old who had a delivery in the previous 24 months from 30 randomly selected *kebeles* (smallest administrative unit in Ethiopia) across Amhara region. The survey included the Self-Reporting Questionnaire (SRQ-20) developed by the World Health Organization—a CMD screening instrument that includes 20 yes/no questions on depression, anxiety, and somatic symptoms experienced in the last 30 days. We used 2 cutoff scores to determine probable cases of mental disorder: (1) 4/5 (≤ 4 “yes” responses = non-case, ≥ 5 “yes” responses = case) based on a study that validated the SRQ-20 against a diagnostic tool in Butajira, Ethiopia, and (2) a more conservative and commonly used 7/8 cutoff.

**Results:**

Among the 1,294 women who completed the full survey including the SRQ-20, 32.8% had probable CMD using the 4/5 cutoff score versus 19.8% using the more conservative 7/8 cutoff. About 15% of the women responded affirmatively that they had had suicidal thoughts.

**Conclusion:**

Poor mental health was common among the surveyed women who had given birth in the past 24 months in Amhara region, Ethiopia. Integrating mental health care into maternal and child health services could potentially alleviate the burden of CMD among women in the extended postpartum period.

## Abstract (precis)

Poor mental health, including suicidal thoughts, affects a substantial proportion of surveyed women who are up to 2 years postpartum in the Amhara region of Ethiopia. Opportunities for integrating basic psychosocial mental health services into maternal and child health services should be explored.

## INTRODUCTION

Mental disorders during and after pregnancy are increasingly recognized for their burden in low- and middle-income countries (LMICs)—for both the disability among the affected women and the associated impact on their children.^1^^–^^4^ A review of studies on postpartum common mental disorders (CMD), which include depression and mixed depressive, anxious, and somatic symptoms, among women in LMICs revealed a weighted mean prevalence of 19.8%.^1^ In Africa, another systematic review found a weighted mean prevalence of 18% for depression among postpartum women and 14% for anxiety.^2^ Risk factors for postpartum CMD include poverty, marital status (unmarried), marital conflict, family conflict, higher parity, adverse reproductive health outcomes (eg, unintended pregnancy, pregnancy complications, stillbirth), and lack of social support.^1^^,^^2^

About 20% of postpartum women in developing countries have common mental disorders.

The magnitude of postpartum CMD in Ethiopia is not well-established. Estimates for depression during and after pregnancy have ranged from 9% to 20%,^9^^,^^10^ and for postpartum CMD, the estimates have been as high as 33%.^11^ Understanding the postpartum CMD burden is important for informing mental health services and policies.

In order to reach postpartum women suffering from disabling depression, anxiety, or somatic disorders, global guidance promotes integrating mental health services into maternal and child health (MCH) care platforms.^5^ In many contexts, integration must extend beyond facility-based services because many women lack access to such services. For example, in the Amhara region of Ethiopia, 89% of women deliver at home and 93% do not receive a postpartum checkup within 41 days after delivery.^6^

To improve access to services for many of the poorest and most remote Ethiopians, the government established the Health Extension Program, which includes a cadre of trained health extension workers (HEWs) who provide community-based services.^7^ Per government guidelines, HEWs are at least 18 years old, are women (except in pastoralist areas), have at least a 10th grade education, and are nominated and selected at the local level.^7^ Each *kebele* (smallest administrative unit in Ethiopia, similar to a ward) has 1 health post (lowest level of primary care) with 2 HEWs who provide a variety of health services, including the pregnancy continuum of care. The HEWs conduct home visits, hold community conversations on health topics, and distribute health-related commodities. In theory, they also have mental health training to screen people for mental health problems and refer them for services, and they are tasked with mental health prevention, promotion, and ongoing community-based care.^8^ Diagnosis and treatment for mental disorders is offered at the next level of care (health centers), staffed by health officers and nurses.

This report describes the mental health status of Ethiopian women up to 2 years postpartum in the Amhara region. The mental health data are from a study undertaken by the Amhara Regional Health Bureau and FHI 360 whose main purpose was to identify factors associated with postpartum family planning use; the study hypothesized that mental health was one such factor but did not find an association between mental health status and postpartum family planning uptake.^12^

## METHODS

A cross-sectional survey was conducted among 1,319 women aged 15–49 years old who had delivered within the previous 24 months, from 30 randomly selected *kebeles* across Amhara region. Participants were recruited from 3 randomly selected zones, comprising East Gojjam (n = 389), South Wollo (n = 385), and North Gondar (n = 390), as well as 1 additional zone, Bahir Dar (n = 130), which was purposefully selected for urban representation. Data collectors approached local health workers, including HEWs, at the selected *kebeles* to generate lists of women who had delivered within the last 24 months regardless of whether the delivery took place at a health facility or at home. In addition, the selected women were asked if they knew any other women who had delivered within the last 24 months in their area.

The survey included the Self-Reporting Questionnaire (SRQ-20) developed by the World Health Organization (WHO), which is a CMD screening instrument that includes 20 yes/no questions on depression, anxiety, and somatic symptoms experienced in the last 30 days.^13^ Each of the 20 questions is scored 0 (no) or 1 (yes), with the total score ranging from 0 to 20; a cutoff score is used to determine probable cases of mental disorder or poor mental health. The SRQ-20 has been used extensively in Ethiopia among various populations as well as across Africa.^10^^,^^14^^–^^16^ For this study, we obtained an Amharic-translated and pretested version of the SRQ-20 from colleagues who used it with a perinatal population.^10^

The WHO Self-Reporting Questionnaire is a simple and commonly used tool to screen for common mental disorders.

We received ethical approval from the Protection of Human Subjects Committee at FHI 360 and the Institutional Review Board at the Amhara Regional Health Bureau. Participants provided oral informed consent. All interviews were conducted at participants' homes (usually outside to ensure confidentiality during the interview). Participants who responded affirmatively to the suicidal ideation item or to ≥ 10 questions were referred to local health services.

A total of 1,294 nonpregnant women who had given birth in the previous 24 months completed the survey including the SRQ-20. We present CMD prevalence estimates using 2 different SRQ-20 cutoff scores. The first is a 4/5 cutoff score (ie, ≤ 4 “yes” responses = non-case, ≥ 5 “yes” responses = case), which we selected based on an unpublished study that validated the SRQ-20 against a diagnostic tool in antenatal clinics in semi-rural areas around Butajira, Ethiopia. The study found an optimal cutoff score of 5 or more with 80% sensitivity and specificity (personal communication with Dr. Charlotte Hanlon, Addis Ababa University, 2014 Apr 2). Another study using the SRQ-20 with perinatal women in Ethiopia found conflicting evidence for optimal cutoff scores.^10^ We therefore also present SRQ-20 data using a 7/8 cutoff, which has been commonly used in other studies around the world.^18^ The bivariate association between mental health and relationship status was conducted using Pearson's chi-square test and adjusted for clustering at the *kebele* level.

## RESULTS

Among the 1,294 surveyed women, 32.8% had probable CMD using the 4/5 SRQ-20 cutoff score (responding affirmatively to 5+ items). Using a more conservative cutoff of 7/8 (responding affirmatively to 8+ items), 19.8% had probable CMD.

20% to 33% of the surveyed women in Amhara region had poor mental health.

Probable CMD by zone using the 7/8 cutoff was 24.1% in North Gondar, 15.2% in East Gojjam, 19.5% in South Wollo, and 21.5% in Bahir Dar.

A sizable proportion of women (n = 187, or 14.5%) responded affirmatively to the question, “Have you thought of ending your life?”, indicating the recent burden of suicidal thoughts among this population (Table).

**Table.** SRQ-20 Results for Probable Mental Disorder/Poor Mental Health in the Past 30 Days Among Women With a Delivery in the Past 24 Months, by Relationship Status, Amhara Region, Ethiopia

**SRQ-20 Questions**	**Total Sample (N = 1,294)**	**Married (n = 1,174)**	**Unmarried (n = 120)**
	No. responding “yes” (%)		
1. Been nervous, tense, or worried	320 (24.8)	271 (23.1)	49 (40.8)
2. Frightened easily	265 (20.5)	236 (20.1)	29 (24.2)
3. Generally felt unhappy	247 (19.2)	203 (17.4)	44 (37.0)
4. Found it difficult to make decisions	149 (11.5)	120 (10.2)	29 (24.2)
5. Had headaches quite often	519 (40.1)	466 (39.7)	53 (44.2)
6. Had problems thinking clearly	204 (15.8)	174 (14.9)	30 (25.2)
7. Found it difficult to enjoy daily activities	186 (14.4)	146 (12.5)	40 (33.3)
8. Often lost interest in things	152 (11.8)	117 (10.0)	35 (29.2)
9. Felt tired easily	402 (31.1)	355 (30.3)	47 (39.2)
10. Experienced loss of appetite	312 (24.2)	287 (24.5)	25 (21.0)
11. Problems with sleep	278 (21.6)	243 (20.8)	35 (29.4)
12. Had uncomfortable feelings in stomach	310 (24.0)	282 (24.1)	28 (23.3)
13. Often experienced shaking of hands	139 (10.8)	126 (10.8)	13 (10.9)
14. Felt tired all the time	359 (27.8)	322 (27.5)	37 (30.8)
15. Cried more than usual	194 (15.1)	159 (13.6)	35 (29.2)
16. Daily activities suffered	140 (10.8)	118 (10.1)	22 (18.5)
17. Thought of ending life	187 (14.5)	154 (13.2)	33 (27.5)
18. Felt unable to play a useful part in life	170 (13.2)	147 (12.6)	23 (19.2)
19. Experienced poor digestion	258 (19.9)	220 (18.7)	38 (31.7)
20. Felt worthless	160 (12.4)	129 (11.0)	31 (25.8)

Abbreviation: SRQ, Self-Reporting Questionnaire.

All variables had ≤ 6 missing responses. Percentages are based on non-missing data.

About 15% of surveyed women had had suicidal thoughts.

Unmarried women (n = 120, or 9.3% of the total sample) were significantly more likely than married women to experience poor mental health (*P* < .0001). In this sample, unmarried women were primarily widowed, separated, or divorced.

Internal reliability of the SRQ-20 was excellent (Cronbach's alpha = 0.92).

## DISCUSSION

This study highlights the substantial levels of mental distress and probable CMD among the surveyed women who had delivered in the past 24 months in Amhara region, Ethiopia, including unmarried women. A recent review of CMD prevalence studies conducted in Ethiopia reveals that depression and the broader category of CMD have been measured with a range of instruments in various populations, making specific contextual comparisons challenging.^19^ Our estimates are somewhat higher than those from other studies in Ethiopia conducted among women of reproductive age and among the general population, suggesting that women up to 2 years postpartum may potentially be at elevated risk of CMD compared with a general population of women of reproductive age.^19^^,^^20^ More research is needed, however, to explore this potential relationship further since several limitations with the study design limit our ability to draw firm conclusions. Furthermore, it is worth reiterating that the SRQ-20 captures a range of mental distress symptoms, more inclusive than depression alone. Nonetheless, it is clear that a substantial proportion of women in this study were experiencing mental health problems, including suicidal thoughts, for which services are urgently needed.

The global health field has called for integrating mental health care into priority health care platforms, and specifically for integrating maternal mental health care into MCH services.^5^^,^^21^ Given the low rates of facility-based postpartum checkups in Amhara region and nationally, access to HEWs for mental health prevention activities and referral for more serious mental health problems during the extended postpartum period is vital. Ethiopia's national mental health strategy mandates the integration of mental health services into the primary health care system, and it emphasizes self-care and use of HEWs for promotion and prevention activities to increase awareness, reduce stigma, and increase use of mental health services.^8^ Given the high rates of probable CMD among women up to 2 years postpartum, mental health services could potentially be integrated into MCH services offered by providers based at local health posts and/or by HEWs, such as at childhood immunization and well-child visits. The possibility of HEWs providing simple psychosocial interventions, such as screening for maternal mental distress, basic counseling, and facilitating establishment of support groups, could be further explored and tested in Ethiopia.^22^

### Limitations

This study used a convenience sample of women identified through local health care providers, which excluded very remote communities that could not be reached by vehicle, and therefore may not be representative of the entire Amhara region. Also, while our focus was on mental distress in the postpartum period, which is usually defined as the first 12 months after childbirth, our sample consisted of women who had delivered within the past 24 months, which limits our ability to associate the mental distress estimates within the conventionally defined postpartum period. Our ability to draw firm conclusions about the occurrence of CMD specifically within the postpartum period is further limited because the study did not include a comparison group of women who had not given birth. However, the sample of women who were up to 24 months postpartum represents an extended postpartum period, and thus the estimates from this study add to the literature on maternal CMD.

## Notes

**Peer Reviewed**
